# Development of the Home Fall Hazard Checklist

**DOI:** 10.1155/2021/5362197

**Published:** 2021-05-31

**Authors:** Christina Ziebart, Neha Dewan, Joshua Tuazon, Joy MacDermid

**Affiliations:** ^1^Department of Rehabilitation Sciences, Faculty of Health Science, Western University, London, ON, Canada; ^2^Department of Health Sciences, Lakehead University, Thunder Bay, ON, Canada; ^3^Temerty Faculty of Medicine, University of Toronto, Toronto, Ontario, Canada; ^4^Physical Therapy and Surgery, Western University, London, Ontario, Canada, Roth∣McFarlane Hand and Upper Limb Centre, St. Joseph's Hospital, London, ON, Canada

## Abstract

**Objective:**

Home hazard assessment is particularly important following a fracture as a means of preventing subsequent fractures. The purpose of this study was to evaluate current checklists and evidence on home hazard to develop a usable self-administered checklist that could be used by adults to assess home hazards.

**Design:**

Review and observational, prospective study. *Setting*. Community dwelling. *Participants*. Nine adults (4 men, 5 women) were asked to review the checklist and provide feedback on whether items were relevant, comprehensive, and easy to understand. *Intervention*. A search for literature examining the causes of falls that focused on home hazards or behaviours was conducted, and causes were extracted. Using the combined list of home hazards, a draft checklist was created. The participants were asked to pilot the checklist through their home. *Primary and Secondary Outcome*. An initial iteration of the checklist was modified to reduce redundancy (by grouping certain items together), improve usability (by adding a “not applicable category”), and improve readability (by removing double-barrelled questions or rewriting certain items).

**Results:**

This process resulted in 74 items in 10 areas. On average, it took 10 minutes for the participants to complete the home walk-through while filling out the checklist.

**Conclusion:**

The fall hazard-home checklist is a new checklist designed to identify home fall hazards with the intended use of being either administered by self-report through memory or supported by a walk-about, and that could potentially be completed by a patient who has incurred a fall, fracture, a family member, or caregiver. Given the expense of home hazard assessments that involve a home visit, the validity of this method of detection warrants further investigation.

## 1. Introduction

One-third of community dwelling adults fall every year [1, 2]. According to the WHO report on global burden of disease, fall-related injuries are the third leading cause of years lived with disability, accounting for 11.6% of deaths in 2013 [2]. In a systematic review, it was noted that history of fall had the highest odds of predicting future falls, particularly in recurrent fallers (OR = 3.46 95%, 95%CI = 2.85-4.22), when comparing to other sociodemographic risk factors [3]. One in three falls leads to serious injuries, including fractures [4, 5].

Distal radius fracture (DRF) is a common, fall-related low-energy fracture among people aging 50+, resulting from falling on an outstretched hand from a standing height or lower [6, 7]. DRF accounts for an estimated lifetime risk of 33% (females) and 6% (males) after the age of 50 [8, 9]. Otherwise, healthy people with DRF demonstrated site-specific bone loss at the distal forearm and generalized low bone mass indicative of a poor bone health state [10–12]. A transition to physical inactivity is reported after DRF [13–16]. The reduced physical activity due to pain, disability, and fear of falling after a fall-related DRF is theorized to increase the risk of future falls and fractures due to subsequent bone loss and decreased effectiveness of protective responses from the deterioration of muscular strength, balance, coordination, and reaction time [14, 17–22]. Meta-analyses conducted in 2000 and 2003 characterized the direct and indirect associations between DRF and subsequent fractures of the spine, wrist, and hip [23, 24]. In a 3-year follow-up of 158,940 postmenopausal women (PMW), it was reported that prior wrist fracture increased future fracture risk three-fold for the wrist and two-fold for osteoporotic (OP) fractures elsewhere. This risk was independent of bone mineral density (BMD) or other common risk factors for OP fractures [25].

Ninety percent of all OP fractures result from a fall [26]. Nearly 50-75% of falls from standing height occur in and around the home or backyard during everyday activities such as walking, turning, stair climbing, and stepping over obstacles [27]. Patients with home hazards are at four times greater risk of falls than those without home hazards [28]. PMW with DRF might have more risk of secondary falls and OP fractures due to more time spent in the home, and physically inactive lifestyles as bone and muscle mass decrease after menopause. This suggests fall risk assessment could be an effective physical therapy strategy to prevent secondary falls and OP fractures among relatively healthy older adults with DRF [29–31].

One strategy to reduce the number of falls in older adults is by detecting and ameliorating fall hazards in the home. Evaluation of home fall hazards can be performed by experienced clinicians conducting a home visit, with or without assistance in ameliorating the hazards identified. These interventions are time-consuming, dependent on the availability of qualified personnel and health system support for these types of activities. With efforts to maintain costs and healthcare systems, such visits are rarely feasible for patients who do not have caregivers coming to the home for other aspects of their care. However, robust evidence from the meta-analysis demonstrates home safety intervention could reduce falls by 39% among at-risk seniors [32]. Home fall hazard checklists are potentially a simple and cost-effective fall prevention strategy. In a traditional home safety assessment, a therapist scans the home with a fall hazard checklist to identify potential hazards [32–35]. Therapist home visits to identify and remediate hazards within the home might be considered one of the gold-standard methods for the prevention of secondary falls and fractures but are rarely feasible [36].

Currently, several fall hazard assessment checklists exist, but with limitations. A review of the literature is presented in the results of this manuscript, going into depth on the strengths and limitations of other fall hazards checklists. We believe there is a need for a checklist, which is substantially comprehensive to be used in routine physical therapy practice for older adults at risk of falls or osteoporotic fractures such as PMW with DRF. Therefore, the goal of this implementation study was to develop a Comprehensive Home Fall Hazard Checklist (CHFHC) and instruction tutorial based on literature synthesis of scientific findings and existing fall hazard programs and to facilitate a comprehensive and efficient patient assessment of home fall hazard for use on secondary fracture prevention following a fragility fracture (DRF).

## 2. Methods

The study was conducted in 3 phases to develop a CHFHC for older adults at risk of a home-related fall. In Phase 1, the literature was examined to identify known hazards and validated checklists. In Phase 2, previous checklists were subject to content analysis and integrated with findings from Phase 1 to develop a draft checklist. In Phase 3, adults were asked to provide feedback to ensure the checklist was comprehensive and clear to understand. Ethics was obtained from the McMaster ethics board for the component of the study involving participants. Participants provided informed consent.

We have used comprehensive methods to draft preliminary/initial version of CHFHC (Appendix A). The CHFHC is a checklist designed to help community dwelling adults navigate their home hazards to reduce the risk of falling. This included (a) review of guidelines for checklist development [37–41] and (b) review of existing checklists for home fall hazard assessment [34–37, 41–46]. The clinicians (physical therapists and occupational therapists) who were experts in assessing patients at risk of fall were requested to provide their feedback on the checklist and instruction manual via an email. A face-to-face team meeting lead by a study team member (ND) was organized by the research team to obtain additional feedback. In this meeting, the checklist along with the instruction manual was presented to the clinician scientists, researchers, and graduate students whose primary focus of research was related with the fall risk screening or older adults. Based on the feedback, we have revised and created a paper version of CHFHC and participant instruction manual.

### 2.1. Phase 1: Literature Review and Data Extraction

Items were generated from a literature search on home-related fall hazard checklists from grey literature, PubMed and CINHAL, in December 2019, without any restrictions to the date. Grey literature included a Google search to identify any potentially relevant current checklists that involve fall hazard identification for adults. The PubMed and CINHAL searches included keywords such as falls, fall hazards, home or indoor fall risks, and checklists.

### 2.2. Phase 2: Development of the Draft Checklist

#### 2.2.1. Checklist Identification and Data Extraction

Several checklists were identified [33–35, 41, 45, 46], and key concepts, such as environmental factors, personal factors, and major themes, in each checklist related to fall hazards were extracted from each checklist. After extracting the data and identifying missing or redundant items, a preliminary version of the CHFHC was developed.

The main categories for the CHFHC were related to environmental factors, as those are common factors related to falls in adults.

#### 2.2.2. Item Pool Evaluation and Reduction

The preliminary version of the CHFHC was made to identify the environmental factors more comprehensively than previous checklists by categorizing potential factors based on the home and surrounding environment. The categories are the driveway and front door, the kitchen, the hallways and living rooms, the bedroom, the bathroom, stairs, laundry room, garage, general, and apparel. In our checklist, we have included some suggestions to keep the home safe and obstacle-free. In addition to the environmental factors, the checklist includes behavioural questions such as “can you easily do this action” to get a sense of difficulty of the tasks. Furthermore, we have evaluated readability of our checklist to ensure that our patient population or their caregivers can use our checklist.

### 2.3. Phase 3: Expert Review and Checklist Revision

When developing a new checklist, it is necessary to ensure that the items are comprehensive and clear. Consistent with the recommendations from Stone, the preliminary version of this tool was circulated to experts in physiotherapy research and clinicians (physiotherapists and occupational therapists) who routinely assess people at risk of falls [47–49]. The experts were asked to evaluate the overall format, domains, and items of the checklist. The experts were comprised of a musculoskeletal researcher with a physiotherapy background, physical therapists working with patients at risk of falls, and patients that may use the checklist. The checklist was revised through iterative feedback. Each domain and item were reviewed for structure and clarity, redundant inquiries eliminated, and ambiguous wording modified.

#### 2.3.1. Patient Review and Checklist Revision

As the final step to develop the checklist, we chose to interview knowledge users (adults at risk of falls) that may use the checklist to identify home fall hazards. A sample of 9 adults (4 men, 5 women) were asked to review the checklist and provide feedback on whether items were relevant, comprehensive, and easy to understand. Adults were asked to review the items of the checklist for clarity and comprehension. Then, the participants were asked to take the checklist home and perform a home walk-through to determine whether items were missing and to ensure that the checklist was comprehensive.

#### 2.3.2. Patient and Public Involvement

The patients and public were involved in refining the checklist. We involved the public by engaging clinicians on the relevant subheadings included in the checklist. The patients were involved in piloting the checklist and providing feedback on the subheadings and usability of the checklist. Patients were not involved in designing the research question or recruitment for the study.

## 3. Results

Overall, the CHFHC underwent 3 rounds of revisions from various expert groups. The first round of revisions was from experts in the field of fall hazard identification and falls prevention. The second round of revisions was from people at risk of falling. The third round of revisions was related to ensuring the document was readable and user-friendly. See the final checklist in Appendix A.

### 3.1. Literature Review: Checklist Identification

#### 3.1.1. The Check for Safety

A home Fall Prevention Checklist for Older Adults has 17 items and is free of cost but currently has no published evidence on usability and effectiveness [42]. The Safe At Home checklist [43] is limited in use to occupational therapists and cannot be used by a patient or caregivers directly.

#### 3.1.2. The Safe Living Guide

A guide to Home Safety for Seniors [44] is a comprehensive checklist that addresses nine different hazard areas such as the outside, inside, stairs, bathroom, and kitchen but is aimed at all populations, not specific for older adults, and does not offer solutions for addressing the identified hazards. Finally, the Home Safety Self-Assessment Tool (HSSAT) [34] is a reliable and valid tool but is 64-item long, missing garage-related hazards, and requires an occupational therapist consultation. Finally, the Home Screen is meant to be a quick home assessment for older adults but requires nurse administration of the checklist [37]. See Table 1 for a review of the strengths and limitations of each checklist.

### 3.2. Checklist Refinement from Experts

Based on the feedback from the therapists and researchers, Drive Way and Front door were included in the same category to avoid redundancy. Apparel was included as a separate item. Items were made more specific. For example, the location of grab bars was mentioned. Instead of using the term step stool, we have changed it to stable step-stool; key concepts were underlined throughout the checklist. Examples and pictures were included to improve clarity and ease the risk factor screening.

### 3.3. Checklist Refinement from People at Risk of Falling

A total of nine older adults were interviewed to gain insight on their experience using the CHFHC, and to provide feedback to ensure the checklist is thorough, clear, and easy to follow.

On average, it took 10 minutes for the participants to complete the home walk-through while completing the checklist, with some participants needing only 5 minutes and one participant taking 20 minutes. Participants were then asked to rate the clarity and ease of use of the checklist on a scale from 1 to 10 with 1 being the least clear and hardest to use and 10 being the most clear and easiest to use. The average score was 9 out of 10, with the lowest score being 8 out of 10 and the highest score being 9 out of 10.

Participants provided specific feedback on items of the checklist. The participants wanted to see a column to select not applicable for certain items. For example, one of the participants lived in an apartment complex, so when asked to evaluate the hazards in his garage, the question was not applicable. To address this comment, we added a line in the instructions to leave the box blank if the question did not apply. One of the participants mentioned that they lived in an older house that had a mudroom. The participant noticed that there were certainly fall hazards in this space, but there was not a specific category addressing it. The participant suggested adding a “comments” section to allow for additional feedback on potential fall hazards. To address this comment, we have provided a blank page at the end of the checklist to allow for additional comments.

Two of the questions were determined to be unclear by the participants. One of the questions was determined to be a double-barrelled question, where participants were asked if they wear shoes inside or outside. The participants commented that they did wear shoes outside, but not inside and did not know how to respond to the question. To address this comment, we have separated the questions to be asking about shoes outside.

Overall, the checklist received good feedback, with the participants expressing that the checklist is thorough and easy to follow. When asked if they would prefer to have a therapist visit the home or use the checklist on their own, most participants expressed that they would prefer the self-reported checklist. The self-reported checklist was preferable because they could conduct it on their own time and maintain privacy.

### 3.4. Readability Grade Levels

Readability levels were calculated for the final version of this checklist. The Flesch-Kincaid Grade level was 4.4 indicating it is easy to read and can be understood at a fifth grade reading level. No additional revisions were made after the readability testing.

## 4. Discussion

This study developed a new checklist to comprehensively identify fall hazards in the home, the Comprehensive Home Fall Hazard Checklist (CHFHC). This checklist assists adults in identifying potential fall hazards by taking them through their home and systematically evaluating the safety of the home. The checklist is divided by a room beginning with the driveway and front door, then the kitchen, hallways and living rooms, bedroom, bathroom, stairs, laundry room, garage, general, and apparel. This checklist has not yet been validated but is designed to be used for adults and, in this study, was evaluated by older adults at risk of falls.

The CHFHC provides a unique and comprehensive self-report tool to assist with the assessment of factors that may be fall hazards for older adults at risk of secondary falls/OP fractures such as PMW at risk of a DRF. It is known that falls are problematic in adults, and one aspect contributing to fall risk is home hazards. In a network meta-analysis by Cheng et al., home hazard assessments and modification reduced the odds of falling by 76% (OR = 0.76, 95%CI = 0.52, 1.11) when compared to usual care [50], suggesting that home hazard assessment alone can contribute to reduced falls risk.

Assessing fall hazards is a strategy to reduce the number of falls experienced by adults. Home hazards were identified as one of the most common causes of falls, with 80% of falls occurring during daily activities such as going to the bathroom, walking through the apartment, walking the dog, returning from the mall, taking of shoes, opening the front door, opening mail, or going on the balcony to check the weather [51]. In a more general analysis of causes of falls, trips were cited as very common [51]. This CHFHC helps people identify risks for trips, helping to reduce the risk of falls.

The home assessment tool is relevant for populations that are community dwelling and at risk of falling. The current study evaluated adults at risk of falling, who are at an increased risk of developing osteoporosis, and having a fracture as a result of falling. However, this checklist could also be applied to older adults in general. Approximately 1 in 3 community dwelling older adults will fall each year [52], and the fall risk increases as age increases [53]. Fall risk further increases for individuals with chronic conditions, such as those recovering from stroke, with Parkinson's disease, or frail older adults who have experienced muscle atrophy [54]. It is important to note that the tool has not yet been validated in these populations, but this would be valuable for a future work.

The comprehensive self-checklist is a timely tool, to assist adults in self-leading and identifying home hazards. Given the current health state with restrictions on home visits due to the global pandemic, this checklist allows for self-initiated or telehealth-supervised assessments of home hazards. Telemedicine has become a global necessity for providing health care to patients [55], and this checklist may be a tool in guiding both clinicians and patients to identify home hazards and reduce the risk of home falls.

## 5. Strengths and Limitations

A strength of this study is that the literature was thoroughly scanned for previous fall checklists and was used to compile a comprehensive, but not repetitive, checklist. This allowed us to capture a more comprehensive checklist. This study also conducted interviews with individuals that used the checklist and provided feedback to ensure the checklist is clear and easy to follow.

However, important limitations should be considered when interpreting this work. The number and type of patients who evaluated the checklist were small. The patients included were not representative of both orthopaedic and neurologic conditions, although, in principle, the checklist should be relevant for both. We used a survey approach rather than a cognitive interviewing approach to evaluate the checklist from the patient perspective, whereas the latter might have provided more insight into how respondents were interpreting items. This checklist has not yet been tested for psychometric properties, so it is not clear whether the checklist is valid or reliable. Validity evaluation should include more in-depth evaluation of content validity, discriminant validity, and ultimately whether the use of the checklist makes a difference in terms of fall prevention. The next steps would be to conduct psychometric properties and assess whether the CHFHC is able to reduce falls in adults. As with any new checklist, there are potential other limitations such as the checklist not being generalizable, not identifying all fall hazards that exist in different types of homes, potential for items to be misinterpreted, and the length of the checklist may deter participation.

## 6. Conclusions

In conclusion, the fall hazard-home checklist is a new tool designed to help adults identify home fall hazards that is easily understood and captures known fall risks from the literature and prior checklists. It may be a useful tool in secondary fracture prevention, particularly for patients not having caregivers come into their home who might perform this assessment during a home visit. The usefulness of this tool should be evaluated prospectively in terms of its ability to support identification of fall hazards and ultimately in fall and fracture prevention.

## Figures and Tables

**Table 1:** Comparison of currently published home fall hazard checklists.

Checklist	Population	Number of items	Strengths	Limitations
The Check for Safety [42]	Older adults	17	Provides suggestions to further prevent falls Provides additional safety tips	No evidence on usability
The Safe at Home Checklist [43]	Older adults	Unknown	Unknown	Administered by occupational therapists Cannot access online
The Save Living Guide [44]	Adults	70	Moderate reliability (0.509)	Administered by occupational therapists
Home Safety Self-Assessment Tool [34]	Adults	64	High content validity (0.98), test-retest reliability (0.97), and interrater reliability (0.89)	Not comprehensive, missing items related to garage Requires occupational therapy consult
Home Screen [37]	Older adults	12	Psychometric properties assessed, with good construct validity and reliability	Administered by community nurses

## Data Availability

There are no additional data available.

## Appendix

The Comprehensive Home Fall Hazard Checklist (CHFHC) is a checklist designed to help community dwelling adults navigate their home hazards to reduce the risk of falling.
